# *Trichoderma atroviride* seed dressing influenced the fungal community and pathogenic fungi in the wheat rhizosphere

**DOI:** 10.1038/s41598-022-13669-1

**Published:** 2022-06-11

**Authors:** Lina Sui, Junhui Li, Joshua Philp, Kai Yang, Yanli Wei, Hongmei Li, Jishun Li, Ling Li, Maarten Ryder, Ruey Toh, Yi Zhou, Matthew D. Denton, Jindong Hu, Yan Wang

**Affiliations:** 1 School of Bioengineering, Qilu University of Technology (Shandong Academy of Sciences), Jinan, 250353 China; 2 Shandong Provincial Key Laboratory of Applied Microbiology, Ecology Institute, Qilu University of Technology (Shandong Academy of Sciences), Jinan, 250103 China; 3 China-Australia Joint Laboratory for Soil Ecological Health and Remediation, Ecology Institute, Qilu University of Technology (Shandong Academy of Sciences), Jinan, 250103 China; 4 School of Agriculture, Food and Wine, The University of Adelaide, Urrbrae, 5064 Australia

**Keywords:** Microbiology, Microbial communities, Fungi

## Abstract

Fusarium crown rot and wheat sharp eyespot are major soil-borne diseases of wheat, causing serious losses to wheat yield in China. We applied high-throughput sequencing combined with qPCR to determine the effect of winter wheat seed dressing, with either *Trichoderma atroviride* HB20111 spore suspension or a chemical fungicide consisting of 6% tebuconazole, on the fungal community composition and absolute content of pathogens *Fusarium pseudograminearum* and *Rhizoctonia cerealis* in the rhizosphere at 180 days after planting. The results showed that the *Trichoderma* and chemical fungicide significantly reduced the amount of *F. pseudograminearum* in the rhizosphere soil (*p* < 0.05), and also changed the composition and structure of the fungal community. In addition, field disease investigation and yield measurement showed that *T. atroviride* HB20111 treatment reduced the whiteheads with an average control effect of 60.1%, 14.9% higher than the chemical treatment; *T. atroviride* HB20111 increased yield by 7.7%, which was slightly more than the chemical treatment. Therefore, *T. atroviride* HB20111 was found to have the potential to replace chemical fungicides to control an extended range of soil-borne diseases of wheat and to improve wheat yield.

## Introduction

Wheat (*Triticum aestivum* L*.*) production is challenged by soil-borne fungal diseases such as Fusarium crown rot and wheat sharp eyespot. Among these, Fusarium crown rot is a disease of global significance that occurs in the major winter wheat growing regions in China^1,2^. It rapidly spread throughout the country in recent years, especially in the HuangHuai area (the main wheat production in China), posing a major threat to the safety and security of wheat production^3–6^. Fusarium crown rot is caused by *Fusarium* spp., amongst which *Fusarium pseudograminearum* is the dominant pathogen^7–9^. Studies have shown that the infection by some *Fusarium* pathogens may directly affect food safety by leading to mycotoxin contamination of grains^10^. In addition to Fusarium crown rot, wheat sharp eyespot caused by *Rhizoctonia cerealis,* is also a major soil-borne fungal disease of wheat^11–14^. The main circumstances that drive infection by the above-mentioned diseases are the annual return of straw to the field, resulting in the accumulation of fungal sources in the soil, and the use of susceptible wheat varieties^5^. The main control methodsare crop rotation, the use of tolerant varieties and biological or chemical agents.

Most of the chemical preparations for controlling wheat diseases use tebuconazole, propiconazole, difenoconazole and/or kresoxim-methyl as active substances, which are fungicides that inhibit the biosynthesis of ergosterol and lead to excessive electrolyte loss^15,16^. Biological agents *Streptomyces*, *Bacillus subtilis*, *Pseudomonas*, *Actinomyces*, *Trichoderma*, etc.^17–24^ can reduce the colonization of pathogenic fungi, produce antibiotics and organic compounds in the soil, and promote plant growth^25^.

Seed dressing is a cost-effective method that has the potential for large-scale application in the prevention and control of crop diseases^26^. Seed dressing with biological agents is widely used to prevent or mitigate fungal diseases caused by pathogenic fungi such as *Fusarium *sp.^27–29^. As a biocontrol agent, *Trichoderma* seed dressing has been increasingly used to control plant fungal diseases. Common pathogens controlled by *Trichoderma* include *Bipolaris sorokiniana*, *F. pseudograminearum*, *F. oxysporum*, *R. solani*, *R. cerealis* and many other plant pathogens^30,31^. Internationally, more than 60% of registered biofungicide formulations contain at least one strain of *Trichoderma*^32^, which is due to the frequent ability of *Trichoderma* spp. to detect, invade and destroy pathogenic fungi^33^. Research has shown that when fungi colonize plant roots, and when different pathogens are detected, the abundance of *Trichoderma* changes^34^. *Trichoderma* spp. can also promote plant absorption of nutrients, plant height, dry and fresh weight of roots and stems, and grain weight^35–37^. At present, commonly used *Trichoderma* species include *T. harzianum*, *T. atroviride* and *T. aculeatus*^38^.

In recent years, the regulation of the structure and interaction of plant microbiomes as a biological control strategy for pathogens has become a focus for research^39,40^. The rhizosphere is a significant plant microbiome for beneficial and harmful microorganisms. Understanding the community compositions of rhizosphere microorganisms and how these changes in response to treatments with biological controls is particularly important in the process of pathogen control. The composition of soil microbial communities can be better understood, and information about pathogens is not required before sequencing with the application of high-throughput sequencing technology^41–44^. Fluorescent quantitative PCR (qPCR) technology can quickly and quantitatively detect target pathogens^45–48^. The combination of the two methods can accurately analyze the changes in rhizosphere microorganism communities and populations of pathogenic fungi.

In this study, high-throughput sequencing and qPCR technology were used to evaluate the biological control effect of *T. atroviride* HB20111 upon the major wheat soil-borne diseases. The work is mainly to quantify (1) the impact of *Trichoderma* seed dressing treatment on wheat fungal communities, and (2) the biological effect of *Trichoderma* in the control of Fusarium crown rot and wheat sharp eyespot.

## Results

### The abundance of fungal pathogens

**Figure 1:**
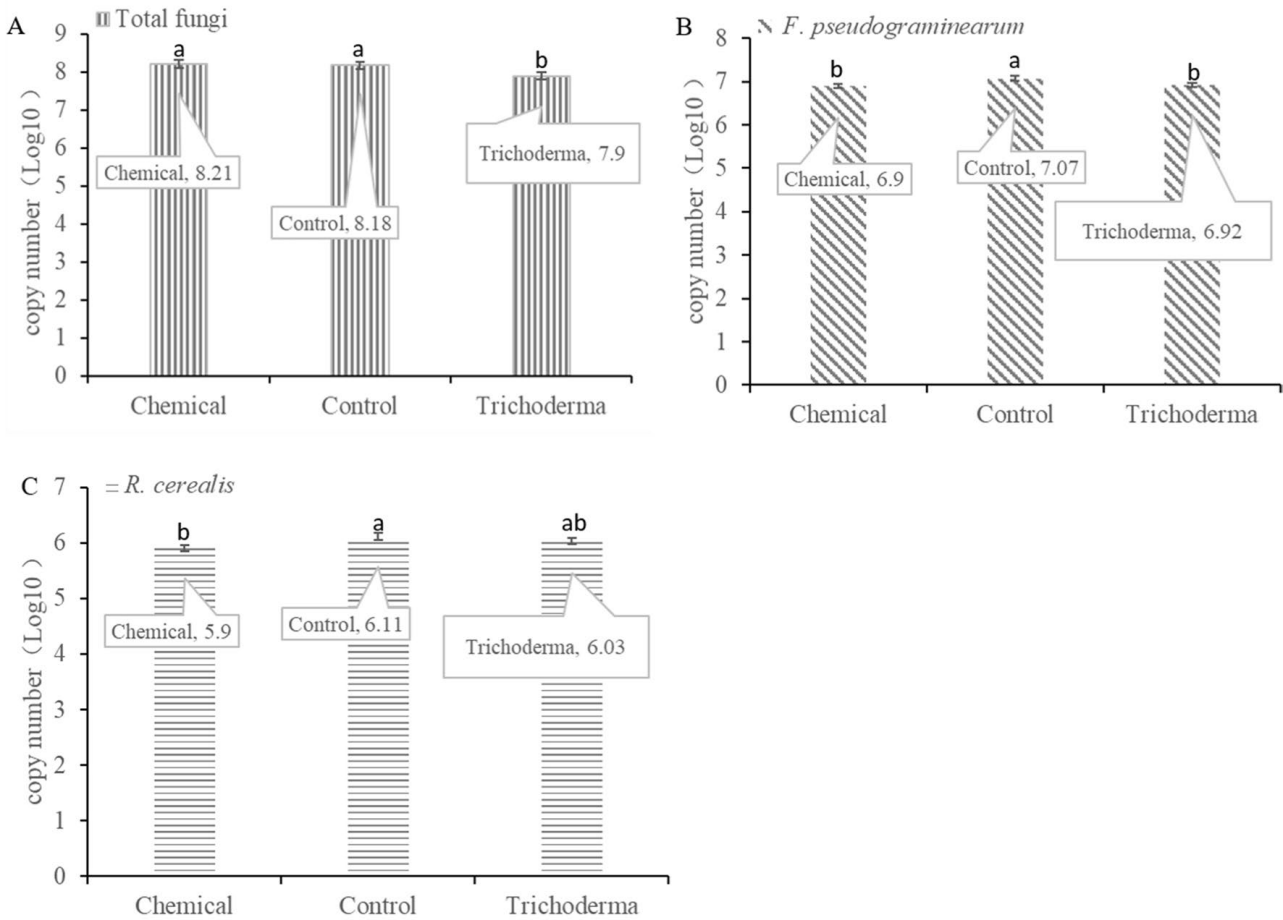
Effects of control seed dressing (Control), chemical fungicide seed dressing (Chemical) and *T. atroviride* HB20111 seed dressing (*Trichoderma*) on the copy number of targeted fungal pathogens. The copy number of total fungi (**A**), *F. pseudograminearum* (**B**) and *R. cerealis* (**C**).

qPCR results showed that different treatments had different effects on the abundance of targeted fungal pathogens (Fig. 1, Supplementary Fig. S1). The copy number of total fungi in *T. atroviride* HB20111 treatment was lower 47.2% than that of the control, in the meanwhile, the chemical fungicide was slightly higher than that of the control, but the differences were not significant (*p* < 0.05, Fig. 1A). With the chemical fungicide and *Trichoderma* seed dressing, the copy number of *F. pseudograminearum* in the rhizosphere of wheat was significantly lower than that of the control with values of 32.5% and 28.9% respectively (*p* < 0.05, Fig. 1B). For *R. cerealis,* the chemical treatment was significantly lower than that of the control by 38.5%, and there was no significant difference in *Trichoderma* treatment (*p* < 0.05, Fig. 1C). The *F. pseudograminearum* and *R. cerealis* levels in the *Trichoderma* treatment were not significantly different from the chemical treatment (*p* < 0.05).

### OTU composition

**Figure 2:**
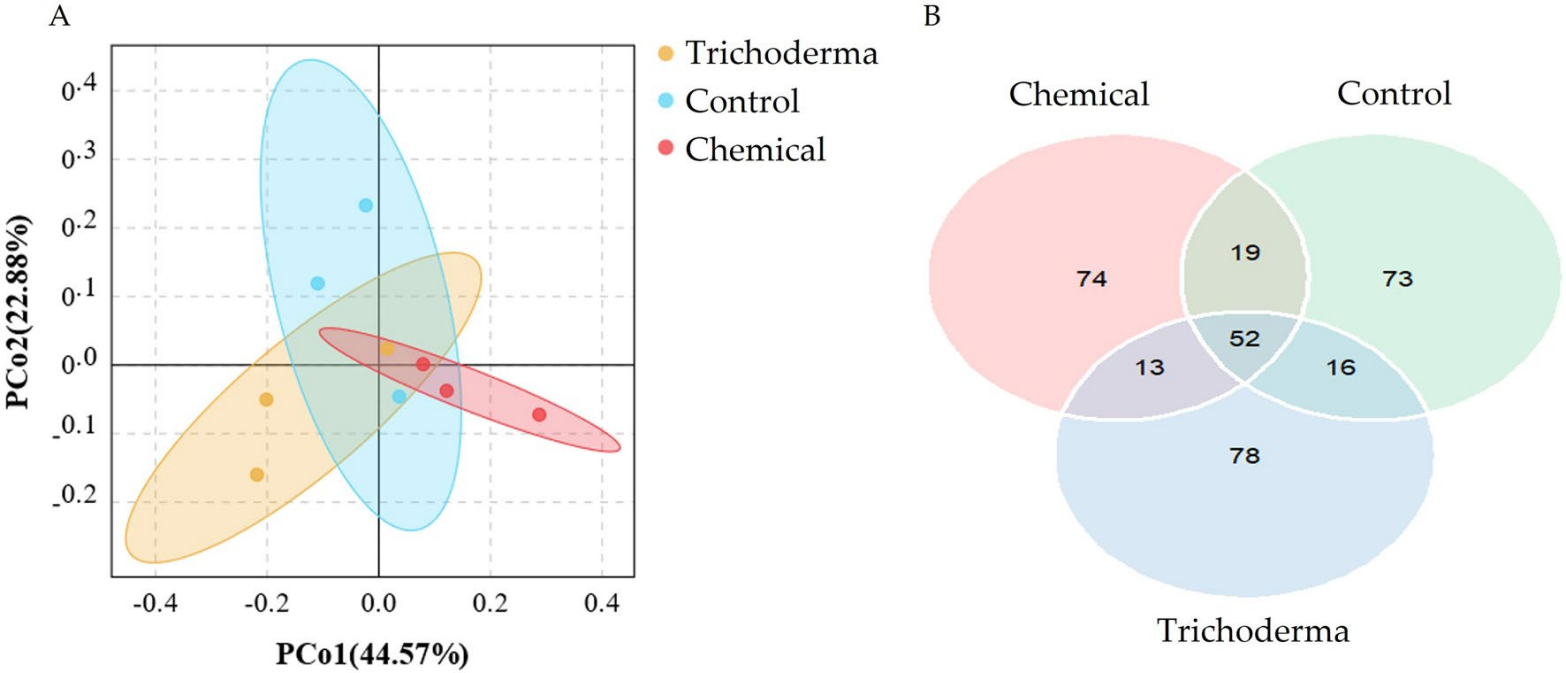
OTU composition analysis of wheat rhizosphere soil under the conditions of control seed dressing (Control), chemical fungicide seed dressing (Chemical) and *T. atroviride* HB20111 seed dressing (*Trichoderma*). Principal component analysis (**A**) and Venn distribution diagram (**B**).

OTU composition analysis was shown in Fig. 2. The first two principal coordinates signified 44.57% (PCo1) and 22.88% (PCo2) of the soil fungal community variation. The X-axis distance between the three different treatment groups were not significant (*p* < 0.05), indicating that *Trichoderma* and chemical treatment did not have a significant impact on the beta diversity of the microbial community. The Venn diagram showed the overlap of OTUs in the microbial community between the different treatments. The total number of OTUs for the three treatments did not change significantly. Among the three treatments, the number of unique OTUs detected by the *Trichoderma* treatment was the most, at 78, accounting for 49.1% of the total, followed by chemical treatment with 74, accounting for 46.8%.

### Abundance and diversity

**Figure 3:**
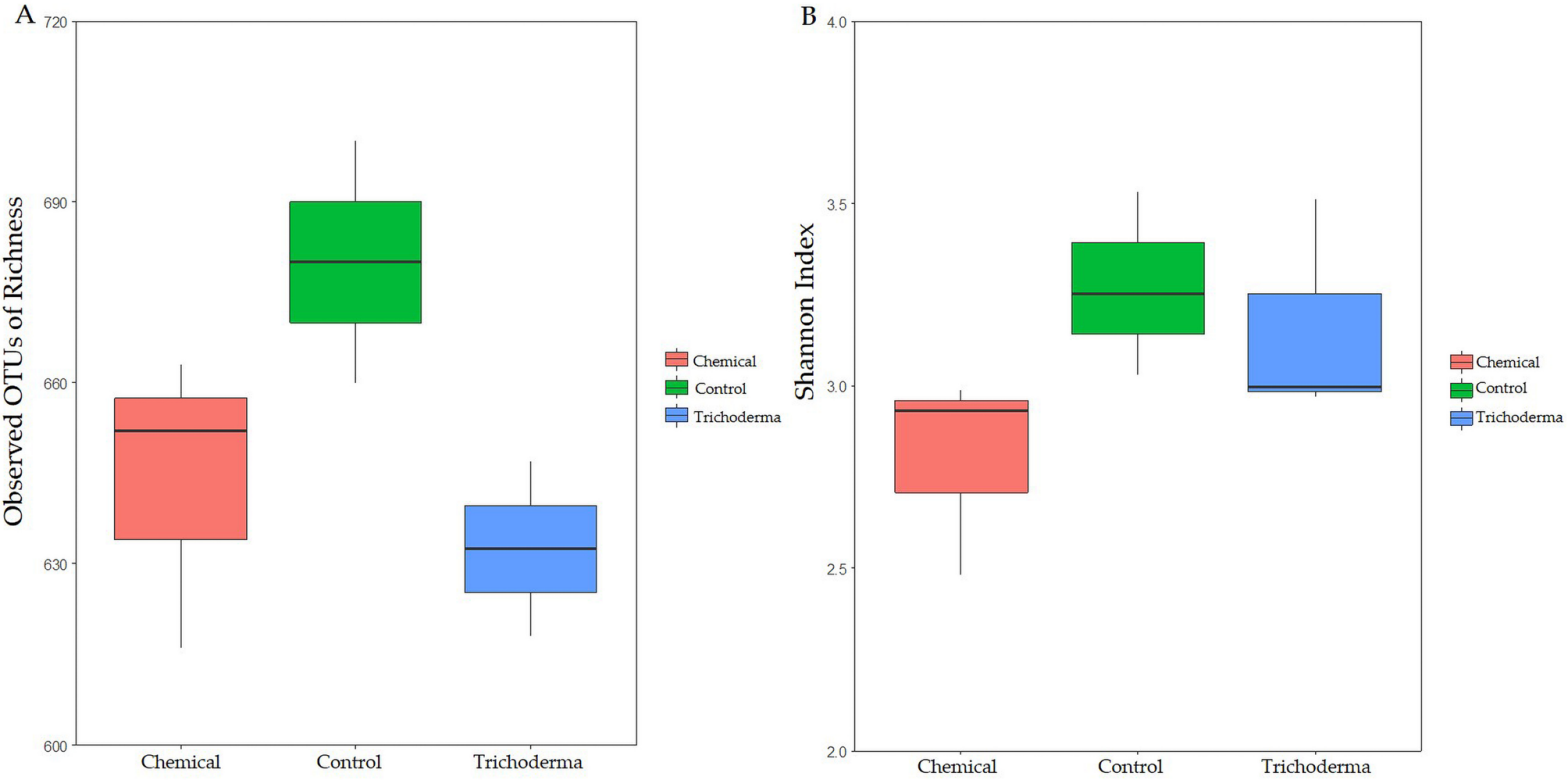
The diversity and richness of the fungal community in wheat rhizosphere soil under different treatments i.e. control seed dressing (Control), chemical fungicide seed dressing (Chemical) and *T. atroviride* HB20111 seed dressing (*Trichoderma*). Observed OTUs of Richness of different treatments (**A**) and Shannon index of different treatments (**B**).

Alpha diversity analyses targeting the fungal community of the wheat rhizosphere showed that both *T. atroviride* HB20111 and chemical fungicide seed dressing treatments affected the structure and composition (Fig. 3). The treated group had slightly less fungal OTUs than that of the control (Fig. 3A), and Shannon index were slighter lower under the chemical and *Trichoderma* treatments (Fig. 3B).

### The structure of the fungal community

**Figure 4:**
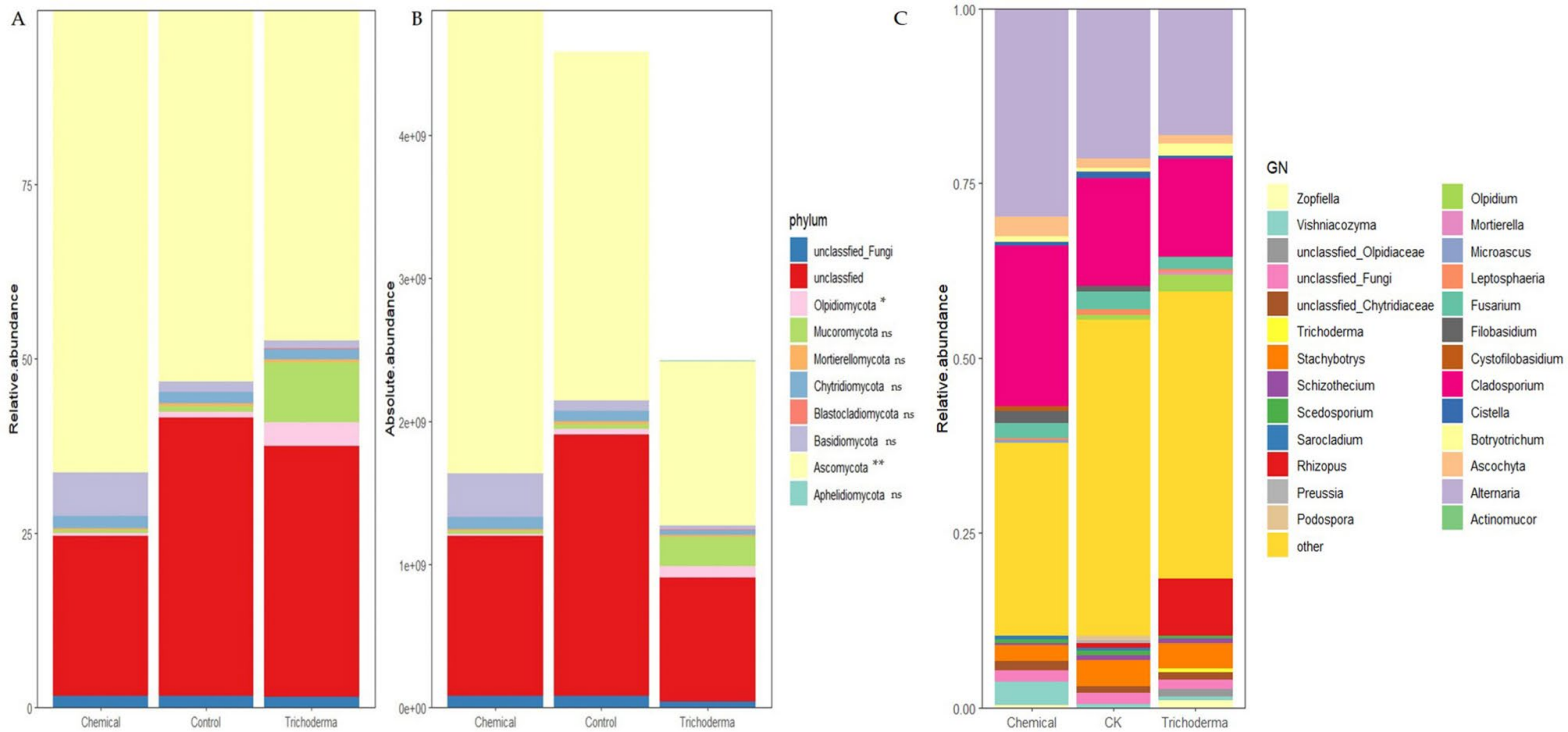
Rhizosphere fungal community structure at the phylum and genus level as affected by control seed dressing (Control), *T. atroviride* HB20111 seed dressing (*Trichoderma*), and chemical fungicide seed dressing (Chemical). Relative fungal abundance at the phylum level (**A**), absolute fungal abundance at the phylum level (**B**) and relative fungal abundance at the genus level (**C**). ns, not significant at *p* < 0.05; *Indicates significance at p < 0.05; **Indicates significance at *p* < 0.01 based on Games–Howell as the post hoc test and Benjamini–Hochberg FDR as the multiple test correction method.

The relative abundance of fungi at the phylum level for each treatment was shown in Fig. 4A. Multiplying the total amount of fungi by the relative abundance to get the absolute abundance (Fig. 4B). The relative abundance of fungi at the genus level for each treatment was shown in Fig. 4C. It can be seen from the relative abundance that different treatments affected the composition of the fungal community (Fig. 4A). Ascomycota was the most dominant phylum, accounting for about 50% in each treatment. The proportion of Ascomycota in untreated rhizosphere soil was 6% higher than *Trichoderma* treatment and 13% lower than that of chemical treatment. The proportion of Mucoromycota and Olpidiomycota in *Trichoderma* treatment were 8% and 3% higher than the other two groups, respectively. In addition, the content of Basidiomycota in the chemical treatment was 5% higher than that in the other two groups (Fig. 4A). And the absolute abundance calculations that the 3 seed dressing treatments had different impacts on wheat rhizosphere soil fungal community composition (Fig. 4B). Compared with the control group, most of fungi decreased in different degrees in *Trichoderma* treatment. Ascomycota and Olpidiomycota were changed significantly in the rhizosphere fungal community following *Trichoderma* seed dressing, compared with the control, among which, the content of Ascomycetes decreased by 54.2%, and the Olpidiomycota increased by 54.8% (*p* < 0.05, Fig. 4B). The dominant genera differed according to treatments (Fig. 4C), except the genera with the highest relative abundance in different treatments were all *Alternaria* spp. and *Cladosporium* spp. *Trichoderma* and chemical treatments both reduced the relative abundance of *Fusarium* spp. The relative abundance of *Alternaria* spp. in the *Trichoderma* treatment decreased by 3.26% and 11.28% compared with the control and chemical treatments, respectively. These decreases were accompanied by notable increases in the relative abundance of *Olpidium* and *Botryotrichum* in the rhizosphere soil. Inoculation caused the inoculant, *Trichoderma*, to have the relative abundance (< 1%), whereas it was not found in other groups.

### Correlation between *Trichoderma* treatment and fungal pathogens in the wheat rhizosphere

**Figure 5:**
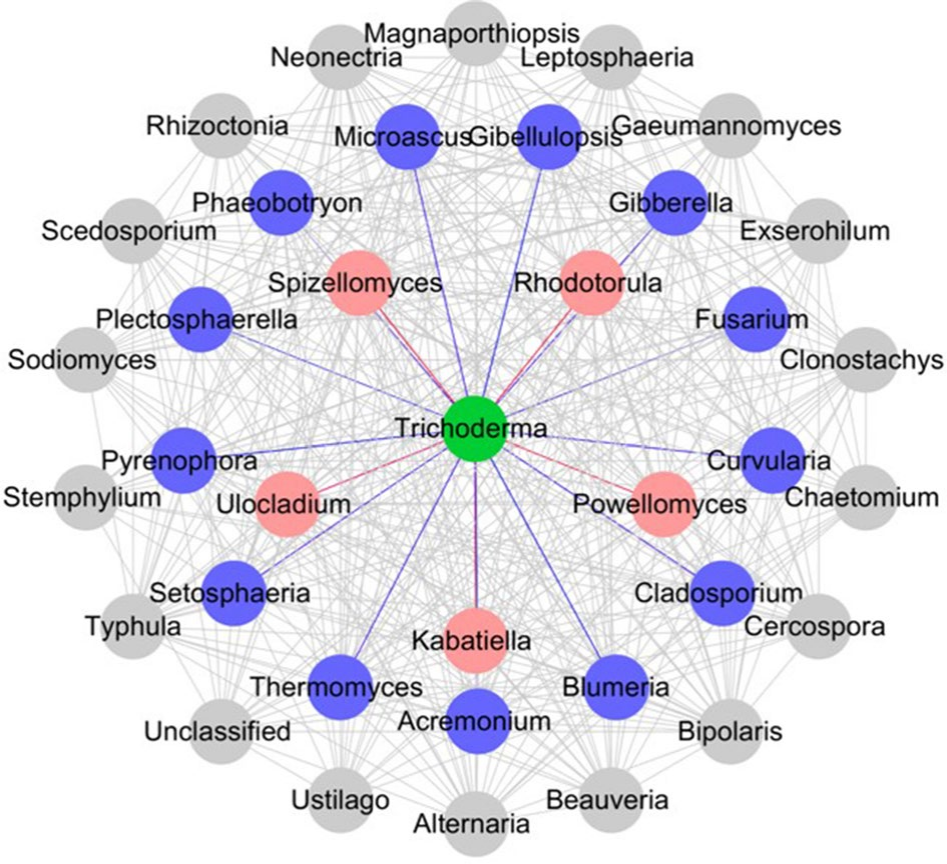
Network diagram of linear relationship between *Trichoderma* and plant pathogens. The red mark represented a positive correlation, and the blue was negative (*p* < 0.05).

Co-occurrence Network Analysis showed that *Trichoderma* had a linear relationship with 18 plant pathogens, of which 13 were negatively related (Fig. 5). The pathogenic fungi that could cause Fusarium crown rot was negatively correlated with the occurrence of *Trichoderma*. In addition to *Fusarium*, *Trichoderma* also had inhibitory effects on many other pathogens.

### Impact of *Trichoderma* on the targeted wheat soil-borne diseases

**Figure 6:**
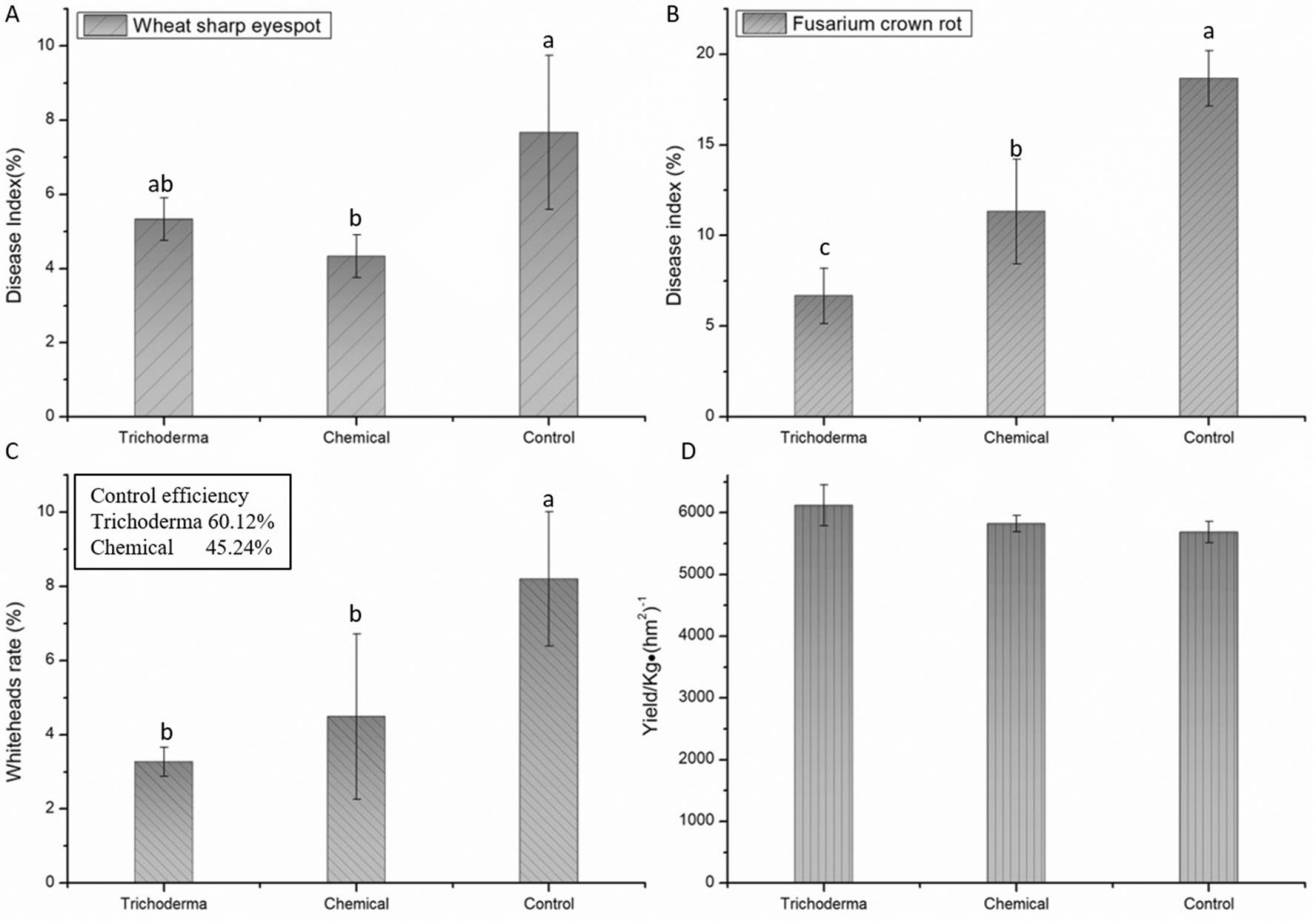
Impact of *T. atroviride* HB20111 seed dressing (*Trichoderma*) and chemical fungicide seed dressing (Chemical) on the targeted wheat soil-borne diseases. The disease index of wheat sharp eyespot (**A**); the disease index of Fusarium crown rot (**B**); the whiteheads rate and control efficiency of different treatments (**C**); the yield of different treatments (**D**).

The survey results of wheat sharp eyespot and Fusarium crown rot disease at 220 days after sowing showed that both *T. atroviride* HB20111 and chemical fungicide seed dressing treatments significantly reduced the disease index of them (*p* < 0.05, Fig. 6A,B, Supplementary Fig. S2). For Fusarium crown rot, *Trichoderma* and chemical treatments reduced the disease index by 64.3% and 39.3%, respectively. We observed that the stalk bases of whiteheads appeared fragile and were mostly covered with brown spots, matching the typical symptoms caused by Fusarium crown rot. The survey results showed that both *T. atroviride* HB20111 and chemical fungicide seed dressing treatments significantly reduced the whiteheads rate (*p* < 0.05). The control efficiency of *T. atroviride* HB20111 seed dressing was 60.1%, compared to the 45.2% control obtained with chemical seed dressing (*p* < 0.05, Fig. 6C). Compared with the control, the yield of wheat treated with *T. atroviride* HB20111 seed dressing increased by 7.7% and with chemical fungicide seed dressing increased by 2.4% (*p* < 0.05, Fig. 6D, Supplementary Fig. S3).

## Discussion

It had been reported that *Trichoderma* could control Fusarium crown rot, most of which were *T. harzianum*^49–51^. We believed that the different effects of *Trichoderma* on the control of wheat diseases may be related to pathogenic fungi, *Trichoderma* strains, ecological environment and rhizosphere soil microbial communities. Studies had shown that *T. harzianum* reduced the disease incidence by 51.79% against Fusarium crown rot which caused by *F. culmorum*^52^. The experimental results showed that *T. atroviride* HB20111 could effectively reduce the occurrence or severity of wheat diseases, including Fusarium crown rot and wheat sharp eyespot, when used to coat wheat seeds (Fig. 6A,B). The control efficiency was as high as 60.1% (Fig. 6C), which was higher than that of a chemical fungicide (45.2%), and may had the added benefit of being able to promote the growth and disease resistance of plants. Seed coating has been widely used in agriculture, and can promote plant health in the soil through early disease pressure and trigger plant defense responses to prolong the effect of biological control of strains^53–55^. This was confirmed by the control efficiency and final output of the field experiment of this work.

In this study, we explored the effect of seed dressing with *T. atroviride* HB20111 on the fungal community in the rhizosphere soil of wheat. OTU composition analysis showed that different treatments did not change the beta diversity of the fungal community, but *Trichoderma* treatment impacted its composition (Fig. 2A). Compared with the control group, alpha diversity analysis showed that both *Trichoderma* and chemical treatment slightly reduced the community abundance, yet the diversity of *Trichoderma* treatment not changing significantly (Fig. 2B). Treatment with *Trichoderma* spp. had been reported to show higher abundance of fungi^56^. Ideally, *T. atroviride* HB20111 inoculation would activate soil microbes and increase their diversity. However, combined with qPCR results analysis (Fig. 1), it indicated that *Trichoderma* treatment reduced the content of some wheat pathogenic fungi so that the species richness decreased, but the community uniformity improved, which was similar to the results of previous studies^57,58^. Owing to the advantages of vigorous vitality and rapid growth, *Trichoderma* spp. can inhibit the growth space of pathogenic fungi by altering the soil microbiome to absorb the necessary nutrients and induce pathogen suppressiveness. Conversely, both the richness and diversity of the fungal microbial community in the rhizosphere of wheat in the chemical seed dressing treatment were both significantly reduced (Fig. 2B). The richness and diversity of microbial populations in the rhizosphere of plants were closely related to plant health^59^. Therefore it can be seen that the *Trichoderma* treatment has multiple advantages in that it does not cause chemical pollution and has less impact on the community of microorganisms compared to chemical treatment.

The results of ITS sequencing at the phylum level showed that Ascomycota was the dominant fungus in the fungal community (Fig. 4A), which was consistent with the previous reports^60,61^. The colonization of *Trichoderma* in the rhizosphere changed the abundance of fungi at phylum level, such as increasing the content of Olpidiomycota and Mucoromycota. *Trichoderma* seed dressing affected the structure and diversity of the rhizosphere microbial community^62–64^, these changes may created a more suitable micro-ecological environment for wheat growth. Interestingly, when we combined the qPCR total fungi results with the relative content, converting the relative abundance of fungal communities in different treatment groups into absolute abundance, we found that the changes of Ascomycota and Olpidiomycota were significant different compared with their relative abundance (*p* < 0.05, Fig. 4B). In particular, the relative abundance of Ascomycota in *Trichoderma* treatment was higher than that in chemical treatment, but the absolute content was lower, which could better reflect the content of pathogenic fungi in soil. This provides an idea for future accurate analysis of high-throughput sequencing data. *Alternaria* spp. as a pathogen, an endophyte and a saprophyte, could cause various plant diseases such as wheat leaf spot, pumpkin seed rot and cumin blight, etc.^65^. As the most dominant genus, the relative abundance of *Alternaria* spp. was reduced in *Trichoderma* treatment (Fig. 4C), indicating that *T. atroviride* HB20111 may also be effective in controlling plant diseases caused by it. Moreover, the content of the main pathogen, *F. pseudograminearum*, was also decreased. The relative abundance of *Olpidium* spp. and *Botryotrichum* spp. increased, but whether the reason for the increase was related to *Trichoderma* regulation was unknown. Chemical treatment significantly reduced the content of *R. cerealis* in the rhizosphere, but there was no significant difference between it and the *Trichoderma* treatment (*p* < 0.05). In terms of the pathogen abundance in the rhizosphere soil of different treatments (Fig. 1C), the content of *R. cerealis* was low (0.9%). The whiteheads were mainly caused by Fusarium crown rot in the 220-day disease investigation, which showed that the occurrence of disease was related to the content of pathogenic fungi in the soil. In addition, we found that *Rhizoctonia* spp. was almost absent in high-throughput sequencing (Fig. 4C), indicating that high-throughput sequencing has inconsistent amplification efficiency for each fungus. Therefore, it is necessary to combine the results of qPCR and high-throughput sequencing for analysis.

As previously reported, *Trichoderma* inoculants to colonize and persisted in the rhizosphere soil for 8 weeks, up to 112 days^66,67^, which explained why the content of *Trichoderma* in the rhizosphere soil obtained at 180 days was very low. *T. atroviride* HB20111 increased yield by 7.7% (Fig. 6D). It was consistent with the previous experimental results that *Trichoderma* could increase wheat yield by 6–11%^68^. Given that the wheat yield of the *Trichoderma* treatment was higher than that of the chemical treatment and the blank control, it can be seen that there is a great potential for the use of *T. atroviride* for the prevention and control of Fusarium crown rot in the future.

There are many plant pathogenic fungi in the rhizosphere fungal community of wheat. We suggested that the presence of these plant pathogenic genera that were positively related to *Trichoderma* was limited, and not enough to cause plant disease, despite being present at elevated levels. Furthermore, there were no reports indicating that these genera of fungi caused serious disease on wheat specifically. By contrast, the main pathogenic fungi that caused plant symptoms in this work were *Fusarium* species. It was evident that *Trichoderma* treatment was negatively correlated with the abundance of a variety of fungal genera that were well-known as pathogens of wheat rhizosphere, especially *Fusarium*. qPCR and high-throughput sequencing results both showed that the coating of wheat seeds with *T. atroviride* HB20111 decreases *Fusarium* spp. content in the rhizosphere. This is consistent with previous studies that *Trichoderma* had an obvious suppression on *F. pseudograminearum* until the wheat matured^69^. It is a further evidence to support the biological control effect of *Trichoderma* on wheat diseases. In addition, *Trichoderma* was negatively correlated with a range of pathogens such as *Gibberella*, *Bulmeria*, *Cladosporium* and *Setosphaeria* (Fig. 5), which may cause plant diseases such as Fusarium head blight, wheat powdery mildew, tomato leaf mildew and maize leaf spot^70–72^. This indicates that *Trichoderma* has more potential and development space in the application of biocontrol.

During the jointing stage, the disease symptoms were not significant, but the symptoms appeared at flowering and filling period. The content of *F. pseudograminearum* in wheat rhizosphere soil of different treatments was determined by qPCR at 180 days, indicating that *Trichoderma* and chemical seed treatment reduced the content of pathogenic fungi. It was associated with reduced incidence of disease, and consistent with the 220-day disease index survey. This method can monitor the pathogenic fungi and realize early warning of disease occurrence to take preventive measures in advance.

## Conclusions

This study indicated that *T. atroviride* HB20111 seed dressing may create a healthier soil micro-ecological environment for the growth of wheat by reducing the content of plant pathogens in the fungal community of wheat rhizosphere, thereby providing evidence for the prevention and control of important diseases and the improvement of wheat yield.

The use of qPCR in this work further explored the effectiveness of *T. atroviride* HB20111 treatment and chemical treatment in reducing the rhizosphere content of pathogenic fungi that cause wheat diseases, including Fusarium crown rot and wheat sharp eyespot. Among them, the rhizosphere populations of *F. pseudograminearum* and *R. cerealis* in the *T. atroviride* treatment and chemical treatment were lower than in the control. This illustrates that *Trichoderma* has the potential to replace chemical fungicides in the control of plant fungal diseases, providing theoretical basis for the future research on the biological control of wheat by *Trichoderma* and the development of green agriculture.

## Materials and methods

### Overview of the test field

The experiment was conducted at the research station of Shandong Luyan Agricultural Seed Co., Ltd. (37°4′ N,116°7′ E; Xixinzhuang Village, Shentou Town, Lingcheng District, Dezhou City, Shandong Province) between October 8th 2019 and June 16th 2020. The experimental field was level, with a soil pH of 7.5 in CaCl_2_, hydrolyzable N content of 31.15 mg/kg, available P content of 38.41 mg/kg, available K content of 168.5 mg/kg, organic matter content of approximately 1.57% and a moderate soil water content. The site was recently used to produce maize, with all plant residues crushed and returned to the field after harvest. Before sowing wheat, potassium sulfate compound fertilizer (N: P_2_O_5_: K_2_O = 17:17:17, Jinzhengda Ecological Engineering Group Co., Ltd., Linyi, China) was applied to the field as the base fertilizer at a rate of 0.6 t/hm^2^, and urea was added as a topdressing at a rate of 0.23 t/hm^2^ (Luxi Chemical Industry Group Co., Ltd., Liaocheng, China). The field was sown with wheat cultivar Jimai 44 (Shandong Luyan Seed Industry Co., Ltd., Jinan, China) at a rate of 0.11 t/hm^2^, using a mechanical seeder. The cultivar Jimai 44 is a high-yielding and high-quality cultivar, yet is sensitive to Fusarium crown rot. The experimental field was divided into plots of 8 × 1.5 m^2^.

### *Trichoderma* strain

*Trichoderma atroviride* HB20111 isolated by Shandong Provincial Key Laboratory of Applied Microbiology was used in the wheat seed dressing experiment. It was deposited in the China General Microorganism Culture Collection and Management Center (CGMCC) under the registration no. CGMCC16963.

### Seed dressing treatment

Seeds were surface-sterilized, soaked for 5 min in an aqueous solution of 0.5% sodium hypochlorite, then rinsed three times with sterile distilled water and air-dried. The *T. atroviride* HB20111 was grown on Potato dextrose agar (PDA, Dingguo, Beijing, China) for ten days and a conidia suspension was obtained as described in a previous study^73^. Briefly, under sterile conditions, *Trichoderma* culture plates were flooded with sterile water and the resulting conidia suspension was filtered through filter paper to separate the conidia from the mycelium. The conidia suspension concentration was adjusted to 2 × 10^8^ CFU/mL. *Trichoderma* spores were applied gradually to continuously rotating wheat seeds at a rate of 100 mL per 10 kg seeds until complete adhesion and absorption to ensure even distribution of *Trichoderma* among the seeds. Chemical fungicide Raxil® seed dressing consisting of 6% tebuconazole (Bayer Crop Science Co., Ltd.; Leverkusen, Germany) applied at the rate of 30 mL per 10 kg seeds, according to manufacturer's recommendation. The blank group was controlled with water at the rate of 10 mL/kg seed.

### Experimental design

The experiment was designed with three seed-dressing treatments and three replications for each treatment. Treatment 1 was a *T. atroviride* HB20111 seed dressing. Treatment 2 was a chemical fungicide. Treatment 3 was a control. The experimental field was divided into 9 blocks, with each treatment randomly distributed with three blocks.

### The effectiveness of *Trichoderma* seed treatment assessed

20 *Trichoderma*-treated wheat seeds were transferred into 500 mL Erlenmeyer flask place with 100 mL sterile water and placed on shaker (120 r/min) for 30 min. The resulting suspensions and their serial dilutions (10^–3^, 10^–4^, 10^–5^) plated onto PDA agar. Colonies of *T. atroviride* HB20111 were counted after 5 days at 25 °C. Finally, we calculated and got the result with the number of effective coats of 5 × 10^5^ CFU/seed.

### Soil sample collection

At 180 days after sowing (jointing stage), 5 randomly selected wheat plants were harvested from each plot, including their whole root systems. The soil sample of each plot was obtained by removing the attached rhizosphere soils from each plant and combined. Each soil sample was then sieved with a 20 mm mesh and packed in a sterile ziplock bag. The test procedure of DNeasy PowerSoil Kit (QIAGEN, Valencia, CA, USA) was applied to soil samples (0.3 g each) to extract the total soil microorganism DNA for subsequent qPCR and high-throughput sequencing analysis.

### Evaluation of the seed dressing treatments

Effects of the *T. atroviride* HB20111 and chemical fungicide seed dressing treatments against *R. cerealis* (wheat sharp eyespot) and *F. pseudograminearum* (Fusarium crown rot) were evaluated in 3 aspects; disease index and percent of whiteheads at 220 days, and wheat yield at 240 days after sowing.

For disease index, 5 randomly selected wheat plants per plot were assessed for each disease. Assessment of wheat sharp eyespot was performed as per the section “Fungicides control wheat sharp eyespot” in GB/T 17,980.108–2004 pesticide field efficacy test guidelines^74^, as Grade 0 = Asymptomatic; Grade 1: brown or obvious sharp eyespot on the outer leaf sheath and the lesion diameter less than 1/2 of the sheath circumference; Grade 2: obvious sharp eyespot on the outer leaf sheath and the lesion diameter greater than 1/2 of the sheath circumference, with the asymptomatic inner sheath; Grade 3: brown or obvious sheath blight spots on the inner sheath and the lesion diameter less than 1/2 of the sheath circumference; Grade 4: obvious sharp eyespot on the inner sheath and the lesion diameter greater than 1/2 of the sheath circumference; and Grade 5: dead.

Fusarium crown rot was assessed using disease classification standards^75^ on the size of browning on the first internode as Grade 0: no browning; Grade 1; 1–25%; Grade 2: 26–50%; Grade 3: 51–75%; and Grade 4: 76–100%.

Disease indices were calculated as follows:

$$\text{Disease} \,  \text{index }(\mathrm{\%}) = \frac{\sum ((\text{number} \,  \text{ of} \,  \text{ diseased} \,  \text{plants} \,  \text{at} \,  \text{ each} \,  \text{stage})\times (relative \,  value))}{(\text{total} \,  \text{ number}  \,  \text{of} \,  \text{plants} \,  \text{under} \,  \text{investigation}) \times (highest \, incidence \, of \,  disease)}\times 100$$

where “stage” means the level of disease severity, and “relative value” means the grade numbers.

We assessed the presence of whiteheads, which is a common symptom shared by wheat sharp eyespot and Fusarium crown rot, manifested as whitish wheat ears, and slender grains or empty husks. The occurrence of whiteheads was investigated in 5 wheat plants for each plot.

The efficiency of control whiteheads by seed dressing treatments *T. atroviride* HB20111 and chemical fungicide was calculated as follows:

$$  \text{Whiteheads} \,  \text{rate }(\mathrm{\%}) = \frac{(number  \, of \, diseased \, plants)}{(\text{total} \,  \text{number}\,  \text{ of} \,  \text{ plants} \,  \text{ under} \,  \text{investigation})}\times 100$$

$$ \text{Control} \,  \text{ efficiency }(\mathrm{\%}) = \frac{  \text{whiteheads} \,  \text{ rate} \,  \text{ of} \,  \text{ control} \,  \text{ group}-\,  \text{whiteheads} \,  \text{ rate} \,  \text{ of }\,  \text{treated} \,  \text{ group}}{\text{whiteheads} \,  \text{rate} \,  \text{of}  \,  \text{control} \,  \text{ group}}\times 100$$

### Analysis of wheat yield

For wheat yield, grains were harvested using a small plot wheat harvester (Delta, Wintersteiger, Beijing, China). Yield for each plot was measured upon harvest. The yield of wheat is calculated according to the actual harvest of wheat yield to remove impurities and a fixed grain water content of 13%.

### Real-time fluorescent quantitative PCR (qPCR) analysis

The abundances of total fungi in wheat rhizosphere soil were determined using the SYBR Green qPCR method. The ITS1F/ ITS2R primers^76^ were used to quantify total fungal abundance (Table 1). A tenfold dilution series of a plasmid containing the 18S rRNA gene of Saccharomyces cerevisiae was used to generate a standard curve (the number of copies of the gene ranged from 3.0 × 10^3^ to 3.0 × 10^7^). The qPCR reaction system (20 µL) included: 10 µL 2 × SG Fast qPCR Master Mix (Sangon Biotech Co., Ltd., Shanghai, China), 20 µM forward and reverse primers each 1 µL, 7 µL molecular biology Grade water and 1 µL of soil sample DNA. qPCR was carried out using a three-step protocol: Pre-denaturation was at 95 °C for 10 min; denaturation was at 95 °C for 30 s, annealing was at 50 °C for 30 s, extension was at 72 °C for 1 min, for 40 cycles. Fluorescence was acquired multiple times in the extended phase of each cycle (72 °C). The dissolution profile was analyzed after PCR amplification to verify the specificity of the amplification. The procedure for the dissolution curve was: 95 °C, 1 min; 56 °C, 1 min; from 56 °C for every 0.5 °C for 10 s, and then continuous increase 89 times (to 95 °C).

**Table 1:** Quantitative PCR primer sequences.

Target group	Primer probe	Sequence (5′–3′)	Product (bp)
Total fungi	ITS1F	CTTGGTCATTTAGAGGAAGTAA	479
	ITS2R	GCTGCGTTCTTCATCGATGC	
*R. cerealis*	RctubF4	CCTAAAGAGTCTGGAGTAAGTC	203
	RctubR4	GCTAGTGCGGTCAATGTATAG	
*F. pseudograminearum*	Fp_TEF1α.2F	AAAAATTACGACAAAGCCGTAAAAA	82
	Fp_TEF1α.2R	ACTCGACACGCGCCTGTTACCC	
	Fp_TEF1α.2P	ACTCGACACGCGCCTGTTACCC	

The abundances of *R. cerealis* in wheat rhizosphere soil were determined using the SYBR Green I fluorescent dye method. The RctubF4/RctubR4 primers^77^ were used to quantify *R. cerealis* (Table 1). A standard curve (gene copy number range) was generated with a tenfold dilution series of a plasmid containing the β-tubulin gene fragment, from 3.2 × 10^2^ to 3.2 × 10^7^. The qPCR reaction system (20 µL) included: 10 µL 2 × SG Fast qPCR Master Mix (Sangon Biotech Co., Ltd., Shanghai, China), 20 µM forward and reverse primers each 1 µL, 7 µL molecular biology Grade water and 1 µL of soil sample DNA. The qPCR was carried out using a two-step protocol: Pre-denaturation was at 95 °C for 180 s, denaturation was at 94 °C for 15 s, annealing and extension were at 60 °C for 30 s, with 40 cycles. The procedure for the dissolution curve was: 95 °C, 1 min; 56 °C, 1 min; from 56 °C for every 0.5 °C for 10 s, and then continuous increase 89 times (to 95 °C).

The abundances of *F. pseudograminearum* was measured using probe-based qPCR. The Fp_TEF1α.2F/2R primers and Fp_TEF1α.2P probe^78^ were used to quantify *F. pseudograminearum* (Table 1). The probe used was double-labeled with 6-carboxyfluorescein (6-FAM) fluorescent reporter dye and Black Hole Quencher® (BHQ) fluorescence quencher. A tenfold dilution series of a plasmid containing the *F. pseudograminearum* tri5 gene was used to generate a standard curve (the number of copies of the gene ranged from 3.3 × 10^3^ to 3.3 × 10^7^). The qPCR reaction system (20 µL) included 10 µL universal TaqMan premix (Sangon Biotech Co., Ltd., Shanghai, China), 2 µM TaqMan probe 2 µL, 20 µM forward and reverse primers each 1 µL, 2 µL DNF buffer, 3 µL molecular biology grade water and 1 µL of soil sample DNA. The qPCR was carried out using a two-step protocol: Pre-denaturation was at 95 °C for 180 s, denaturation was at 94 °C for 15 s, annealing and extension were at 60 °C for 30 s, with 40 cycles. The procedure for the dissolution curve was: 95 °C, 1 min; 56 °C, 1 min; from 56 °C for every 0.5 °C for 10 s, and then continuous increase 89 times (to 95 °C).

The qPCR reaction procedure was performed using an iCycler iQ5 (Bio-Rad, California, USA) real-time PCR instrument. Each sample was repeated three times, and water was used as a negative control to assess contamination during operation. The copy number of DNA was calculated according to the standard curve.

Standard plasmids were obtained by ligating specific fragments into the pMD18-T vector (Sangon Biotech Co., Ltd., Shanghai, China). A standard curve, based on threshold cycles (Cq), was created for each of the three recombinant plasmids using the corresponding primer pairs (Table 1).

Plasmid copy number calculation:

$$Copy \, number/\mu L=\frac{6.02 \times {10}^{23}\left(copies \, per \, mole\right)\times DNA \, amount(g {\mu L}^{-1})}{\text{DNA} \,  \text{length}\left(\mathrm{bp}\right)\times 660(\mathrm{g\,}{mol}^{-1}{base}^{-1})}$$

All primers used in this study were synthesized by Sangon Biotech Co., Ltd., Shanghai, China.

### Illumina MiSeq sequencing of fungal ITS genes

We selected 9 DNA samples (3 treatments × 3 replicate samples) for fungal community analysis. In the first amplification reaction, primers with ITS3 (CCAGCASCYGCGGTAATWCC) and ITS4 (ACTTTCGTTCTTGATYRA)^79^ were used for ITS rDNA amplification, at 0.2 µmol/L primer. This reaction was prepared in a final volume of 30 µL, containing 15 µL of 2 × Taq master Mix (Thermo, New York, NY, USA), 1 µL each of primers (10 µmol/L), and 20 ng of template DNA. Amplification conditions were: 94 °C for 3 min, 5 cycles of amplification of 94 °C for 30 s, 45 °C for 20 s, 65 °C for 30 s, and 20 cycles of amplification of 94 °C for 20 s, 55 °C for 20 s, 72 °C for 30 s, and 20 cycles of amplification, followed by extension at 72 °C for 300 s. The second round of amplification used Illumina bridge PCR compatible primers, with the first round of PCR products as templates. The reaction system was the same as above. Amplification conditions were: 95 °C for 30 s, 95 °C for 15 s, 55 °C for 15 s, 72 °C for 30 s, 5 cycles of amplification, and 72 °C extension for 300 s. After the amplification was completed, the product was purified and quantified with Qubit2.0. According to the measured DNA concentration, the samples were mixed in equal proportions and homogenized to form a sequencing library, which was sequenced on an Illumina MiSeq sequencer by Sangon Biotech Co., Ltd., Shanghai,China.

### Bioinformatics analysis

The original data were uploaded to the GSA (Genome Sequence Archive) database to submit and save the original information for sequencing (Login number: CRA003983). The ITS sequences were divided into operational taxonomic units (OTUs) based on the similarity between the sequences, and performs bioinformatics statistical analysis at a similarity level of 97%. Unite^80^ database was used was used to obtain OTU table with species classification information by blast comparison.

We deleted plant-derived sequences, resulting in 431,514 readings from 9 samples (average of 47,946 readings per sample). In order to obtain an equivalent sequencing depth for later analysis, all samples in the OTU table were parsed into 42,376 sequences.

### Statistical analysis

All statistical analyses were performed using the IBM SPSS 20.0 software program (IBM Corporation, New York, USA). Data were analyzed using one-way analysis of variance (ANOVA) with Duncan’s multiple range test, and significant differences were identified with the least significant difference test at *p* < 0.05 (HSD_0.05_). The FUNGuild^81^ database was used to identify fungal OTUs belonging to phytopathogenic fungi and to calculate their relative abundance. R’s vegan software package was used to calculate the alpha diversity index (Shannon index and Observed Richness of OTUs). R’s ggplot2 software package was used to plot principal coordinate (PCoA) analysis. The species annotation results at the phylum level of the OTU table are classified and summed, and the average value is calculated to obtain the community composition map of the fungal community. The number of OTUs associated with predicted phytopathogenic fungi in wheat rhizosphere soil under different treatments was analyzed using MUNA^82,83^. Cytoscape (3.6.1) was used to generate co-occurrence networks.

### Ethics approval

The experimental research and field studies on plants, including the collection of plant material, complied with relevant institutional, national, and international guidelines and legislation. The appropriate permissions and/or licenses for collection of plant or seed specimens were obtained for the study.

### Ethics declarations

This article does not contain any studies with human participants or animals performed by any of the authors.

## Supplementary Information

Supplementary Figures.

## Data Availability

The datasets generated during and/or analyzed during the current study are available in the GSA (Genome Sequence Archive) database (http://gsa.big.ac.cn/) with the login number of CRA003983.
