# Supplementary material for: Trichoderma atroviride seed dressing influenced the fungal community and pathogenic fungi in the wheat rhizosphere
Source: Sci Rep. 2022 Jun 11;12:9677. doi: 10.1038/s41598-022-13669-1 (PMC9188553; doi:10.1038/s41598-022-13669-1)
Supplement: Supplementary file 1 — Supplementary Figures. [file 41598_2022_13669_MOESM1_ESM.pdf]

Supplementary Figures

Trichoderma atroviride seed dressing influenced the fungal community and pathogenic fungi in the wheat rhizosphere

Lina Sui[1,2] Junhui Li[2,3], Joshua Philp [3,4], Kai Yang[2,3], Yanli Wei[2,3], Hongmei Li[2,3], Jishun Li [2,3], Ling Li[2,3], Maarten Ryder [3,4], Ruey Toh [3,4], Yi Zhou [3,4], Matthew D. Denton [3,4], Jindong Hu [2,3,]& Yan Wang [1*]*

**hujd@sdas.org (J.H.); qingyuanwangyan@163.com (Y.W.)*

1 School of Bioengineering, Qilu University of Technology (Shandong Academy of Sciences), Jinan 250353, China

2 Shandong Provincial Key Laboratory of Applied Microbiology, Ecology Institute, Qilu University of Technology (Shandong Academy of Sciences), Jinan 250013, China

3 China-Australia Joint Laboratory for Soil Ecological Health and Remediation, Ecology Institute, Qilu University of Technology (Shandong Academy of Sciences), Jinan 250013, China

4 School of Agriculture, Food and Wine, The University of Adelaide, Urrbrae 5064, Australia

Treat	R. cerealis(ng).	Copy.number/uL	Copy.number/uL
CK	0.000807577	258424.6677	405543.7565
CK	0.001557086	498267.4855	
CK	0.00087733	280745.4667	
CK	0.000975591	312189.1355	
CK	0.001388923	444455.4395	
CK	0.001010482	323354.1003	
CK	0.001331726	426152.3018	
CK	0.001519693	486301.823	
CK	0.001937511	620003.3881	
Trichoderma	0.000875589	280188.5567	350518.3812
Trichoderma	0.000896711	286947.5409	
Trichoderma	0.000883125	282600	
Trichoderma	0.001814875	580759.8977	
Trichoderma	0.001515183	484858.6792	
Trichoderma	0.000465957	149106.2587	
Trichoderma	0.001029909	329570.7571	
Trichoderma	0.001037505	332001.4538	
Trichoderma	0.001339476	428632.2865	
Chemical	0.000757543	242413.785	249540.4057
Chemical	0.000832328	266345.0837	
Chemical	0.001005239	321676.6398	
Chemical	0.000554907	177570.3218	
Chemical	0.000821524	262887.6941	
Chemical	0.001025638	328204.1044	
Chemical	0.000674219	215750.226	
Chemical	0.000455147	145646.9041	
Chemical	0.000891778	285368.8924	
Treat	Fp(ng)	Copy.number/ul	Copy.number/uL
CK	0.010860765	3584052.329	3573934.707
CK	0.010960156	3616851.365	
CK	0.010531267	3475318.232	
CK	0.00981105	3237646.344	
CK	0.011590801	3824964.208	
CK	0.011218627	3702146.766	
CK	0.011087374	3658833.442	
CK	0.012025868	3968536.586	
CK	0.00938504	3097063.093	
Trichoderma	0.006445737	2127093.121	2542090.317
Trichoderma	0.006838152	2256590.224	
Trichoderma	0.007113996	2347618.708	
Trichoderma	0.007127137	2351955.332	
Trichoderma	0.007358709	2428373.853	

Trichoderma	*0.008337002*	*2751210.629*	
Trichoderma	*0.007517296*	*2480707.756*	
Trichoderma	*0.008868909*	*2926739.843*	
Trichoderma	*0.009722798*	*3208523.383*	
Chemical	*0.006697936*	*2210318.871*	*2413079.078*
Chemical	*0.005800571*	*1914188.365*	
Chemical	*0.006481761*	*2138981.153*	
Chemical	*0.007779644*	*2567282.638*	
Chemical	*0.008305454*	*2740799.932*	
Chemical	*0.007894706*	*2605252.927*	
Chemical	*0.007566881*	*2497070.798*	
Chemical	*0.007713276*	*2545381.016*	
Chemical	*0.007571018*	*2498436*	
Treat	*Fungi(ng)*	*Copy.number/uL*	*Copy.number/uL*
CK	*0.144932944*	*43479883.2*	*45786583.9*
CK	*0.140546341*	*42163902.3*	
CK	*0.146657088*	*43997126.3*	
CK	*0.150217412*	*45065223.7*	
CK	*0.153044687*	*45913406.1*	
CK	*0.154745696*	*46423708.9*	
CK	*0.161151116*	*48345334.9*	
CK	*0.159688509*	*47906552.8*	
CK	*0.162613723*	*48784116.9*	
Trichoderma	*0.070589635*	*21176890.4*	*24175478.5*
Trichoderma	*0.079871121*	*23961336.2*	
Trichoderma	*0.079731841*	*23919552.4*	
Trichoderma	*0.084226211*	*25267863.3*	
Trichoderma	*0.083311791*	*24993537.4*	
Trichoderma	*0.082761248*	*24828374.5*	
Trichoderma	*0.081590836*	*24477250.7*	
Trichoderma	*0.080056518*	*24016955.4*	
Trichoderma	*0.083125154*	*24937546.2*	
Chemical	*0.168044332*	*50413299.6*	*48833059.6*
Chemical	*0.180621371*	*54186411.3*	
Chemical	*0.177764991*	*53329497.2*	
Chemical	*0.160252378*	*48075713.5*	
Chemical	*0.155660925*	*46698277.4*	
Chemical	*0.159180711*	*47754213.2*	
Chemical	*0.165227602*	*49568280.7*	
Chemical	*0.154483783*	*46345134.8*	
Chemical	*0.143755696*	*43126708.7*	

Figures S1. Effects of different treatments on the copy number of the total fungi, F. pseudograminearum and R. cerealis.

Fusarium crown rot	*Trichoderma*			*Chemical*			*CK*		
1	*1*	*0*	*1*	*0*	*0*	*0*	*1*	*1*	*1*
	0	*0*	*0*	*0*	*2*	*0*	*0*	*1*	*3*
	0	*1*	*0*	*0*	*1*	*0*	*1*	*0*	*1*
	0	*1*	*0*	*0*	*0*	*0*	*0*	*1*	*0*
	0	*0*	*2*	*0*	*0*	*0*	*0*	*0*	*0*
2	*0*	*0*	*1*	*1*	*1*	*1*	*1*	*0*	*0*
	0	*0*	*0*	*0*	*2*	*0*	*0*	*0*	*0*
	0	*0*	*2*	*2*	*0*	*2*	*1*	*1*	*1*
	0	*1*	*0*	*1*	*0*	*0*	*1*	*2*	*1*
	0	*0*	*0*	*0*	*0*	*2*	*1*	*1*	*1*
3	*0*	*0*	*0*	*0*	*1*	*0*	*0*	*1*	*0*
	0	*1*	*0*	*0*	*0*	*0*	*1*	*1*	*0*
	0	*0*	*0*	*2*	*1*	*1*	*2*	*3*	*0*
	0	*1*	*1*	*1*	*1*	*0*	*0*	*0*	*1*
	0	*0*	*0*	*0*	*0*	*1*	*0*	*1*	*1*
4	*0*	*0*	*0*	*0*	*0*	*2*	*1*	*0*	*1*
	0	*1*	*0*	*0*	*1*	*1*	*1*	*1*	*1*
	0	*1*	*0*	*0*	*0*	*1*	*0*	*0*	*0*
	1	*0*	*0*	*0*	*0*	*0*	*0*	*0*	*1*
	1	*0*	*0*	*0*	*0*	*0*	*0*	*1*	*1*
5	*0*	*0*	*0*	*0*	*1*	*0*	*1*	*0*	*0*
	0	*0*	*0*	*0*	*1*	*0*	*2*	*1*	*1*
	1	*0*	*0*	*1*	*0*	*1*	*1*	*1*	*2*
	1	*0*	*1*	*0*	*0*	*1*	*1*	*2*	*1*
	0	*0*	*0*	*0*	*1*	*0*	*1*	*0*	*2*
total	*5*	*7*	*8*	*8*	*13*	*13*	*17*	*19*	*20*
Disease index (%)	*6.67±1.52c*			*11.33±2.89b*			*18.67±1.53a*		
Wheat sharp eyespot	*Trichoderma*			*Chemical*			*CK*		
1	*1*	*0*	*1*	*0*	*0*	*0*	*1*	*1*	*1*
	0	*1*	*0*	*0*	*0*	*0*	*0*	*1*	*0*
	0	*0*	*0*	*0*	*1*	*0*	*1*	*0*	*1*
	0	*0*	*0*	*0*	*1*	*0*	*0*	*1*	*0*
	1	*0*	*0*	*0*	*0*	*0*	*0*	*0*	*0*
2	*1*	*0*	*1*	*0*	*0*	*1*	*1*	*0*	*0*
	0	*1*	*0*	*0*	*0*	*0*	*0*	*1*	*0*
	0	*0*	*0*	*1*	*0*	*0*	*0*	*0*	*0*
	0	*0*	*0*	*0*	*0*	*0*	*0*	*0*	*0*

	1	*0*	*0*	*0*	*0*	*0*	*0*	*0*	*1*
3	*0*	*0*	*0*	*0*	*0*	*1*	*0*	*0*	*0*
	0	*1*	*1*	*0*	*0*	*0*	*0*	*0*	*0*
	0	*0*	*0*	*0*	*1*	*1*	*1*	*0*	*1*
	0	*1*	*1*	*1*	*1*	*0*	*0*	*0*	*0*
	0	*0*	*0*	*0*	*1*	*0*	*0*	*1*	*0*
4	*0*	*1*	*0*	*0*	*0*	*0*	*0*	*0*	*0*
	0	*0*	*0*	*0*	*0*	*1*	*1*	*0*	*0*
	0	*1*	*0*	*0*	*0*	*0*	*0*	*0*	*0*
	0	*0*	*0*	*0*	*0*	*0*	*0*	*0*	*0*
	0	*0*	*0*	*0*	*0*	*0*	*1*	*1*	*0*
5	*0*	*0*	*0*	*1*	*0*	*0*	*1*	*0*	*1*
	0	*0*	*0*	*0*	*0*	*0*	*0*	*0*	*0*
	0	*0*	*0*	*1*	*0*	*0*	*1*	*1*	*0*
	1	*0*	*1*	*0*	*0*	*0*	*1*	*0*	*1*
	0	*0*	*0*	*0*	*0*	*0*	*1*	*0*	*0*
total	*5*	*6*	*5*	*4*	*5*	*4*	*10*	*7*	*6*
Disease index (%)	*5.33±0.58ab*			*4.33±0.58b*			*7.67±2.08a*		

Figures S2. Disease Index of Fusarium crown rot and Wheat sharp eyespot

	Actual yield $kg \bullet (hm^2)^{-1}$	*Impurity rate*	*Water content*	*Final output* $kg \bullet (hm^2)^{-1}$	
Trichoderma	*7248*	*0.0213%*	*12.4%*	*6304.7*	*6119.4*
Trichoderma	*7022*	*0.0096%*	*13.6%*	*6108.4*	
Trichoderma	*6834*	*0.011%*	*13.2%*	*5945.1*	
Chemical	*6756*	*0.0176%*	*12.1%*	*5876.8*	*5822.2*
Chemical	*6893*	*0.0093%*	*12.9%*	*5996.3*	
Chemical	*6430*	*0.0094%*	*13.2%*	*5593.4*	
CK	*6302*	*0.0113%*	*14.1%*	*5482.5*	*5683.3*
CK	*6457*	*0.0086%*	*12.7%*	*5617.2*	
CK	*6840*	*0.0106%*	*12.4%*	*5950.2*	

Figures S3. Final yield of wheat. The moisture content is calculated according to 13%.
